# Using a Chord Diagram to Visualize Dynamics in Contraceptive Use: Bringing Data Into Practice

**DOI:** 10.9745/GHSP-D-19-00205

**Published:** 2019-12-23

**Authors:** Amy Finnegan, Saumya S. Sao, Megan J. Huchko

**Affiliations:** a IntraHealth International, Chapel Hill, NC, USA.; b Duke Center for Global Reproductive Health at the Duke Global Health Institute, Durham, NC, USA.; c Evidence Lab at the Duke Global Health Institute, Durham, NC, USA.

## Abstract

We applied a novel approach to visualizing contraceptive histories from the Demographic and Health Surveys (DHS) contraceptive calendar to elucidate patterns of contraceptive switching and discontinuation (e.g., “churn” in contraceptive use across 2 points in time). Taking the contraceptive calendar from the 2014 Kenya DHS, we used R, an open source statistical programming platform, and the *chorddiag* package to create interactive chord diagrams to visualize contraceptive use trajectories, including switching and discontinuation, for all contraceptive types queried in the DHS. We present screenshots from the interactive version. We also tested the usefulness of our chord diagram with potential users, including family planning researchers and advocates. Chord diagrams are visually appealing and provide users with the ability to investigate unique patterns in contraceptive discontinuation using publicly available data from the DHS. After receiving a brief orientation to a chord diagram, users found the chord diagram easy to understand and manipulate. The chord diagram is a potentially powerful way for family planning researchers, advocates, and program managers to visualize women’s contraceptive trajectories and provides insights into the churn in contraceptive use between 2 discrete time periods.

## Abstract (precis)

A chord diagram is an innovative tool that can be used to visualize switching and quitting in contraceptive use between 2 discrete time points. It complements existing analysis of contraceptive failure rates and provides a richer understanding of contraceptive discontinuation and method switching that can lead to fresh insights to improve family planning programs.

## INTRODUCTION

In low-income countries, one-third of women who initiate a modern method of contraception discontinue within the first year, and one-half discontinue within the first 2 years, potentially putting them at risk for unintended pregnancies, maternal morbidity, and mortality.^1^^,^^2^ Discontinuation rates from hazard models describe the magnitude of the problem, but lack detail on the pathways women take after quitting or switching methods. A better understanding of the reasons for and patterns in contraceptive discontinuation may help identify intervention points to ensure that women who do not desire pregnancy have access to contraceptive methods that meet their family planning needs.

Family Planning 2020 (FP2020) aims to enable an additional 120 million women and girls to use contraceptive methods by 2020. The impact of this ambitious goal will be attenuated if high numbers of women who begin using methods later discontinue. In fact, an estimated 38% of women with an unmet need for family planning are former users of contraception.^3^ This phenomenon of discontinuers has been labeled the “leaky bucket”^4^—even when new users begin contraception many of them later quit and total users may decrease. Although some level of contraceptive discontinuation is anticipated based on individual preferences, a better understanding of the rates and reasons for discontinuation among women who do not desire pregnancy will help to more effectively address unmet need.

The Demographic and Health Survey (DHS) collects data on contraceptive discontinuation through the contraceptive calendar module that was first included in DHS surveys conducted between 1988 and 1991, starting with the second wave. The contraceptive calendar is a retrospective monthly reporting of contraceptive use, births, and reasons for discontinuation over the last approximately 5 years from the date of the survey. Although these data provide a more detailed picture of contraceptive behavior, in their raw form, they can be difficult to navigate without advanced data analysis skills. Family planning advocates, program planners, and practitioners could benefit from more explanatory data to develop programs and advocacy campaigns directed to increase the use of contraceptive methods and meet the FP2020^5^ and Sustainable Development Goals.^6^

The objective of this article is to describe the use of a chord diagram, a novel data visualization technique, to elucidate contraceptive trajectories among users of contraception as captured through the DHS contraceptive calendar. Ultimately, better visualization can lead to better understanding of “churn” and the “leaky bucket” and contribute to programs that meet women’s need for effective family planning.

Better data visualization can contribute to programs that meet women’s needs for effective family planning.

## DATA AND METHODS

### Data

DHS surveys provide a cross-sectional snapshot of a country’s population of women of reproductive age that is representative at the national, subnational, urban, and rural levels.^7^ The Standard DHS is typically collected in 5-year intervals. The DHS has been conducted in 77 countries since the first wave in 1985 until 2017. Beginning in 1990, 65 countries (85%) have included the contraceptive calendar for a total of 168 surveys. Surveys that contain calendars can be identified using the DHS Application Programming Interface and the *rdhs* package in R.^8^

Each Standard DHS survey contains demographic information about women, including their level of education, marital status, fertility preferences, and contraceptive use. For the contraceptive calendar, women are asked to report their contraceptive use and pregnancy status for each month for the 5 years before the survey. Enumerators anchor contraceptive use to events such as the birth of a child or pregnancy. Women who say they have stopped using a contraceptive method are asked to give the reason for discontinuation according to predefined categories. The DHS calendar relies on retrospective reporting, which can lead to underreporting of contraceptive use if use occurred further back in time and may differ by whether a method is user-dependent, such as the pill, or not user-dependent, such as an intrauterine device.^1^^,^^9^^,^^10^

This article uses the contraceptive calendar from the 2014 Kenya DHS, which included 31,079 women of reproductive age (15–49). Approximately 50% of these women (n=14,741) responded to the “long questionnaire” that included the contraceptive calendar. Although use of another survey could present interesting patterns, we chose the most recent survey from Kenya because the country has made commitments to increase access to contraceptives for women, especially in vulnerable areas, and has a moderate rate of unmet need for contraception (17.5% according to the most recent DHS).^7^

### Methods

We created event files from the DHS calendar data where each row represented a person-month. Although we created the event files using R,^11^ an open source coding platform, the DHS has created a comprehensive contraceptive calendar tutorial that has code for creating event files in Stata and SPSS.^12^ These event files can be read into R and used to create the diagrams we describe in this article. To identify new episodes of contraceptive use after non-use, we created a subset of data to include only contraceptive use that was reported after 1 month of non-use. For example, in January 2014, a woman may have reported using the pill. She was included in the sample if she reported non-use of contraception in December 2013, the month prior. We then created a subset of data to include only the person-months during the first month of use and 12 months later. We used this data to create a matrix that shows transitions in contraceptive use between the first month of reported use (baseline), any reported use after a month of non-use that occurred during the 5-year survey period, and 12 months after an episode of use after 1 month of non-use aggregated across all common trajectories between baseline and 12 months. Only women with reported values at both baseline and 12 months were included in the sample. Women who never reported contraceptive use and those who reported discontinuing a method of contraception because they wanted to become pregnant were excluded so that the focus was on women who quit or switched methods while desiring to avoid becoming pregnant. We included all other reasons for discontinuation in the visualization described in this article, but others could choose to exclude them depending on their research question. We used individual sampling weights provided by the DHS.

**FIGURE 1.**
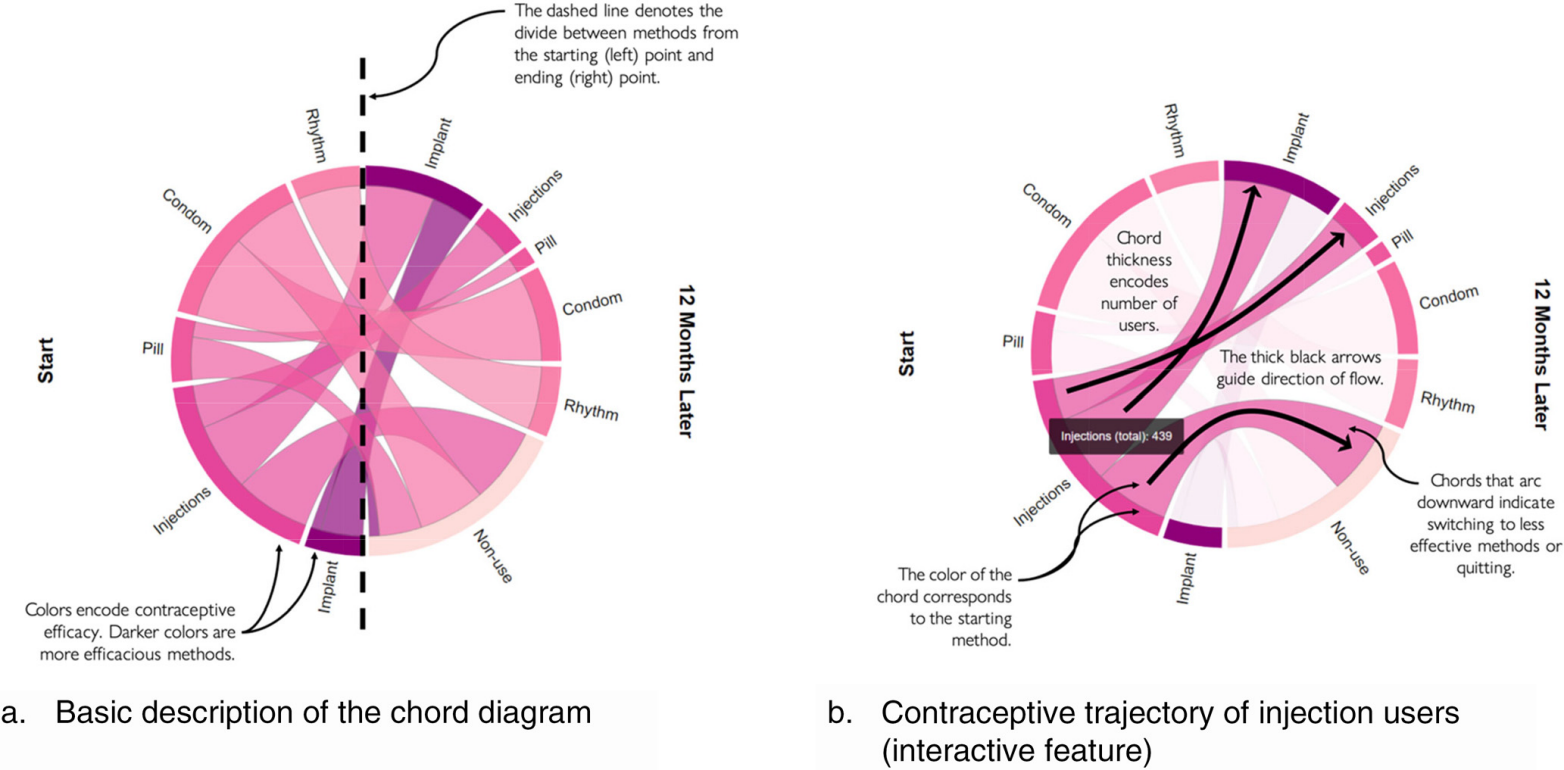
How to Read a Chord Diagram Showing Contraceptive Use This figure displays a chord diagram using hypothetical data on contraceptive use at 2 time points: baseline (start) and follow-up (12 months later). The dashed line (Panel a) splits the chord diagram between the 2 time points. The interactive feature of the same chord diagram (Panel b) is shown, with a focus on women reporting injection use at baseline. Arrows and dotted lines are added for demonstration purposes and are not present in the actual chord diagrams.

We visualized these trajectories using the *chorddiag* package^13^ in R to create an interactive chord diagram that we describe here using static screen shots to show the interactive features. A chord diagram is a circular visualization of interrelated data akin to a transition matrix (shown in the Table), with states represented along arcs and flow between states represented as chords. To visualize contraceptive trajectories from the DHS, we organized the circular chord diagram into halves using the “bipartite” option in the *chorddiag* package. A tutorial of how to read a bipartite chord diagram is shown in Figure 1 using the hypothetical population of women shown in the Table. The beginning period is shown on the left half of the circle, and the ending period is shown on the right half of the circle dissected by a dashed line (panel (a) of Figure 1). The starting population and ending population contain the same number of women so the circle is split directly in half by the 2 periods. Chord diagrams encode the size of flows from 1 period to the next by the width of each chord connecting the starting period to the subsequent analysis period. In Figure 1, the chord size represents the number of women who used each method at baseline and follow-up (i.e., 12 months later). The same hypothetical group of women are displayed in the Table. Focusing on injection users (row 2), there were 439 total injection users at baseline. One hundred women who used injections at baseline were still using injections 12 months later, 161 switched to implants, and 178 quit using injections and weren’t using any method.

**TABLE.** Data Matrix of Hypothetical Contraceptive Use Dynamics

**Method Used at Baseline**	**Method Used 12 Months Later**					
	**Implants**	**Injections**	**Pill**	**Condom**	**Rhythm**	**Non-use**
Implants	100					24
Injections	161	100				178
Pill			36			100
Condom				202		158
Rhythm					148	

Source: Hypothetical data created for tutorial. See Figure 1 for the chord diagram visualization.

A chord diagram is a circular visualization of interrelated data with states represented along arcs and flow between states represented as chords.

The chord diagram (Figure 1) has several features that make it easier to see patterns not immediately obvious in the Table. In the chord diagram, contraceptive methods are organized in order of effectiveness with typical use^14^^,^^15^; darker colors indicate higher efficacy (see panel (a) of Figure 1). In the interactive version, the viewer can use the mouse to hover over a contraceptive method to highlight specific flows to or from that method over time and see both the number of women and the directionality. The color of the chord is set to match the starting period so the viewer can easily see the direction of flows. Panel (b) of Figure 1 illustrates that chords that arc downward indicate women who switched to a less effective method or discontinued use of any method, and chords that arc upward indicate women who switched to a more effective method. If women continued their initial method, the chord draws a line the size of the non-switching population to the same method on the right-hand side of the circle.

We chose the *chorddiag*^13^ package over other R packages because of its ability to work seamlessly “out of the box” for our purposes and others. Other packages to create Sankey diagrams,^16^ another type of flow visualization developed to track energy flows out of systems,^17^ did not allow us to keep the order of contraceptive methods by efficacy and instead placed the bars where they created the least amount of overlap. Likewise, *chorddiag* package versions that created net flow visualizations (the “directional” option) that showed each contraceptive method once around the circumference of the chord diagram and displayed net in and outflows from each state on the same arc were ultimately too confusing to understand compared to “bipartite” chord diagrams used in this article.

The idea for a contraceptive trajectory visualization was pilot-tested at a large, family planning research NGO based in Durham, NC, among staff working directly with contraceptive use data, including the contraceptive calendar from the DHS. After receiving feedback on the need for an interactive visualization tool, the research team developed an early prototype of the visualization using data from the 2014 Kenya DHS, the 2014–2015 Rwanda DHS, and the 2014 Guatemala DHS (these countries were chosen because their survey periods occurred the most recently and overlapped) and debuted it at the International Conference on Family Planning (ICFP) 2018 in Kigali, Rwanda. The pilot-testers were able to interact with the web-based tool and provided feedback on whether the chord diagrams were easy to interpret, showed interesting patterns, and would be of use to the family planning community.

## RESULTS

### Contraceptive Use Dynamics

**FIGURE 2.**
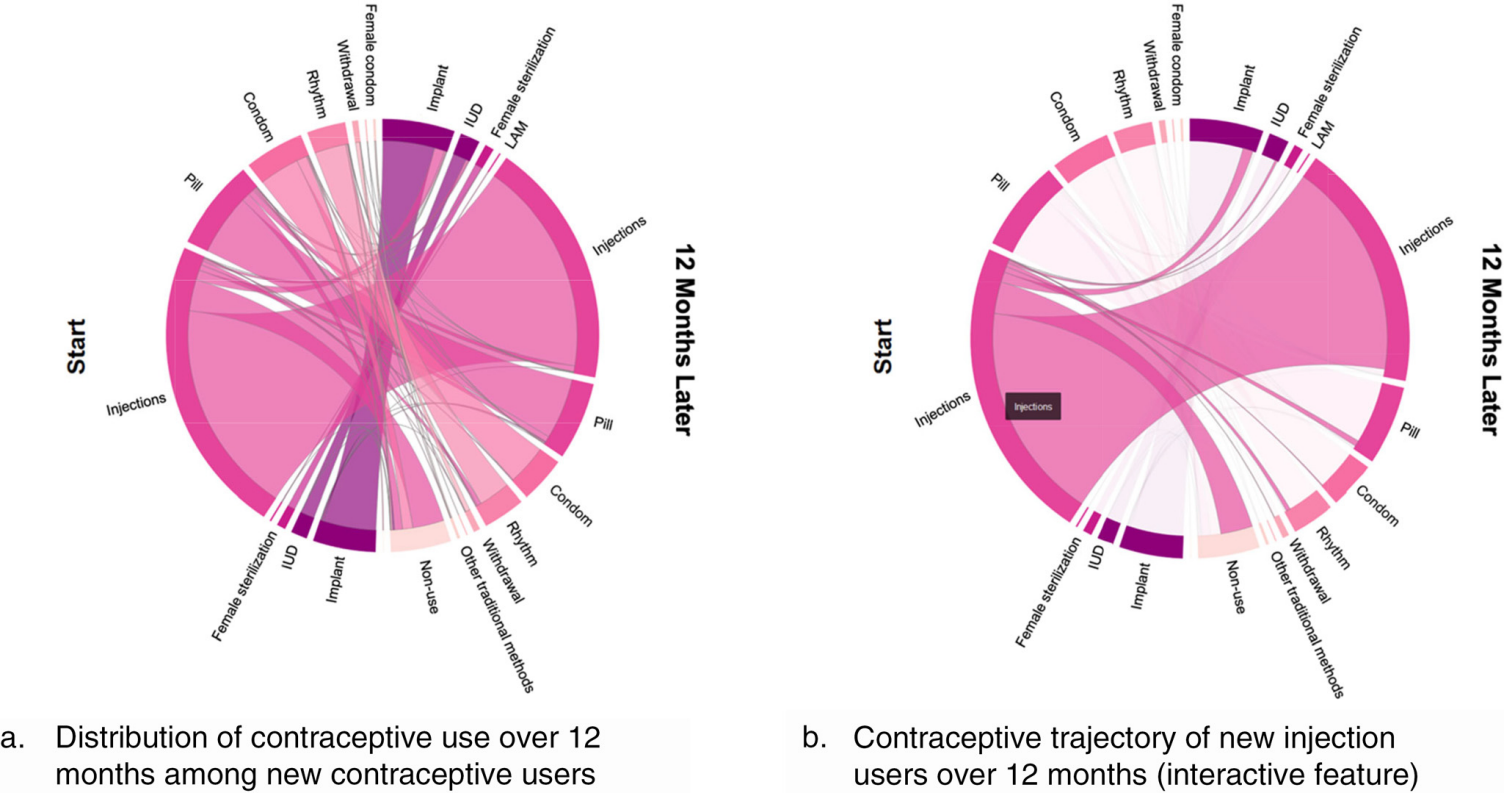
Trajectories of New Contraceptive Users Among Women Sampled in the 2014 Kenya Demographic Health Survey The start period (left) begins with a woman’s first reported use after non-use of contraception in the prior month. The right side (12 months later) displays the method she was using, if any, 12 months later. This population of women mostly uses injections between the 2 time periods (Panel a). Most women who quit using injections either switch to less effective methods or stop using contraception altogether. The trajectories of injection users specifically is shown (Panel b). A few women switch to more effective methods, but most stay on injections. Among those who are no longer using injections 12 months later, most have quit using any method of contraception.

We applied chord diagrams to visualize 12-month contraceptive trajectories for women surveyed in the 2014 Kenya DHS contraceptive calendar. The chord diagram in Figure 2 shows the contraceptive use patterns of the 3,783 women in the Kenyan DHS contraceptive calendar who reported new use of a method of contraception (e.g., use of any method of contraception after 1 month of reported non-use). The aggregated contraceptive trajectories between baseline and 12 months are shown in Panel (a) of Figure 2. The size of each chord around the circumference represents the number of users of each method, weighted by the DHS sampling weight. The arc for injections is the largest because they were the most frequently used method, though they were not the most effective method available indicated by their medium dark color. The light-colored region indicating methods with low efficacy grew from baseline to 12 months, showing that transitions from injections tend to be toward less effective methods or, more typically, non-use. Panel (b) of Figure 2 shows a screen capture of a user hovering the mouse over injection users at baseline. Note that although most users were still using injections 12 months later, as indicated by the thick chord connecting one-half of the chord diagram to the other (the same color as the injection arc), more of those who stopped using injections chose methods that were less effective or stopped using contraception (chords that arc downward) than chose methods that were more effective than injections (chords that arc upward).

**FIGURE 3.**
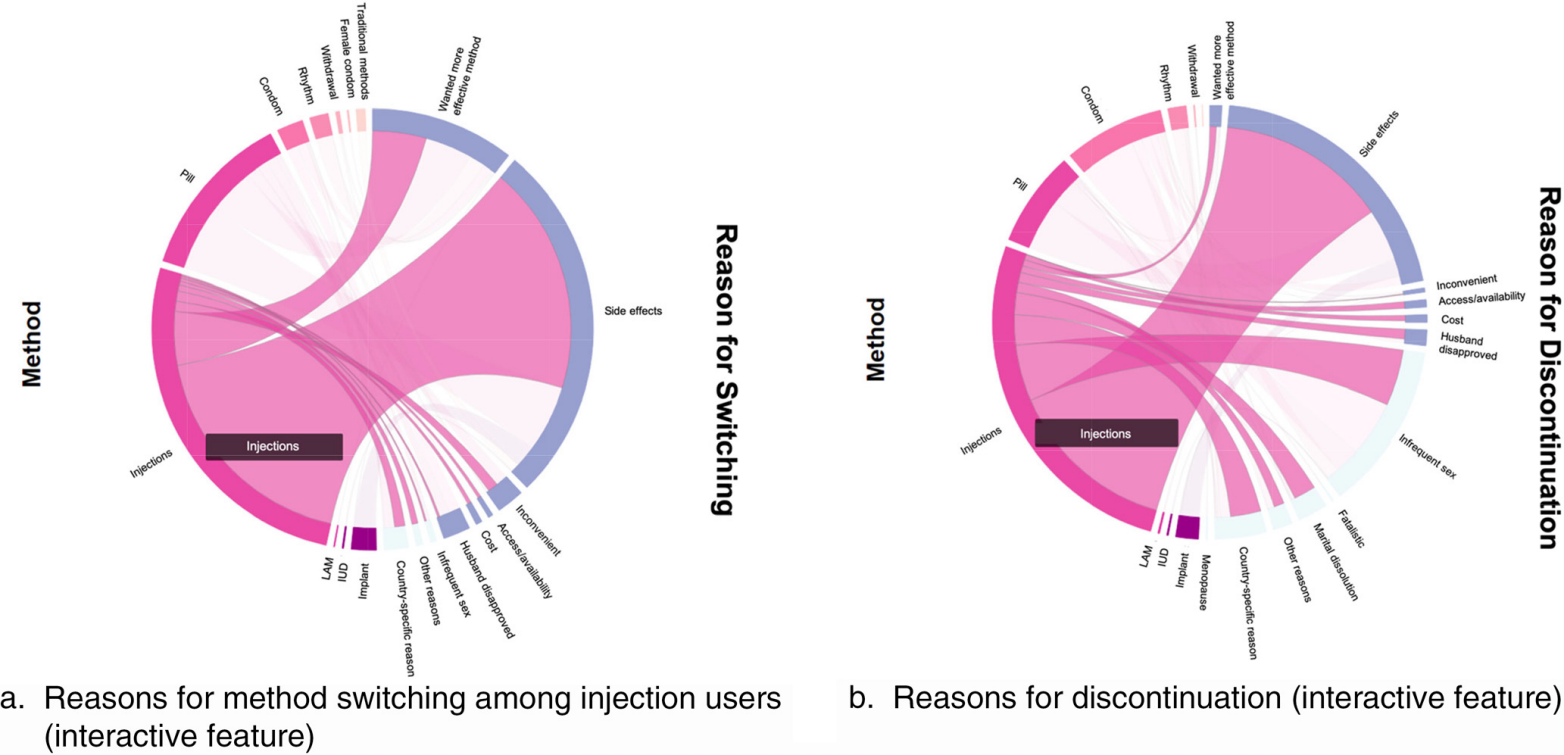
Reasons for Contraceptive Method Discontinuation Among Women Sampled in the 2014 Kenya Demographic Health Survey These chord diagrams display the reasons for switching to another method (Panel a) or discontinuing (Panel b). Colors along the left-hand side encode method effectiveness. The colors along the right-hand side encode reasons for discontinuation into “in need” (dark blue) and not “in need” (light blue). In both cases, the most common reason women quit using injections was because of side effects (both quitting and switching). Comparing Panel (a) to (b), about half of women who quit using any method were still in need while women who switched were still in need.

Chord diagrams can also be used to visualize contraceptive quitting and switching to elucidate the reasons for contraceptive use behaviors. We generated a chord diagram to visualize the reasons that women either switched to another method (Figure 3, Panel a) or discontinued use of a method (Figure 3, Panel b). Figure 3 visualizes reasons for discontinuation or switching among the subset of women using any method at baseline but no method or a different method at the 12-month follow-up. The focus of the example interactive feature is again on women who were using injections at baseline. Recall from Figure 2 panel (b) that most women continued using injections at 12 months, and that most women who stopped using injections quit using contraception altogether rather than switch to another method. Panel (a) of Figure 3 shows that among women who switched from injections to some other method, most did so because of side effects. Cost, access, and inconvenience comprised a small portion of reasons for switching. Panel (b) of Figure 3 shows that nearly all of the women who stopped using injections and did not start using another method did so because of side effects. Comparing reasons for switching (a) to reasons for non-use (b) revealed that most women who switched did so for volitional reasons, including wanting more effective methods or methods with fewer side effects. Discontinuation also was not heavily influenced by cost, access, and convenience suggesting that these reasons may not have been as large a contributor to discontinuation as method-specific reasons.

Chord diagrams can be used to visualize contraceptive quitting and switching to elucidate the reasons for contraceptive use behaviors.

### User Testing

User testing revealed that family planning researchers and practitioners were eager to utilize these chord diagrams as an innovative visualization of DHS contraceptive calendar data. Users commented on the importance of using visualization tools to allow for development of new and more nuanced inferences about contraceptive “churn” from 1 time period to the next among a population of contraceptive users to meet the FP2020 goals. Users who had previously seen a chord diagram present data on migration without 2 distinct spheres (not showing left vs. right or baseline vs. endline)^18^ or other types of flow diagrams (e.g., Sankey diagrams^17^) questioned the value added of the type of chord diagram that showed flows between 2 periods. However, nearly all users were unfamiliar with chord diagrams and were eager to learn more about chord diagrams that could showed flow between 2 periods. These responses demonstrated that the chord diagrams may require a brief tutorial or instructive animation to orient users who are new to this visualization method, which is the motivation for this article. After users were provided with such a demonstration, they were able to easily navigate the chord diagram independently.

Family planning researchers and practitioners were eager to use these chord diagrams as an innovative visualization of DHS contraceptive calendar data.

## DISCUSSION

We found that visualization of contraceptive use, switching, and discontinuation as trajectories (e.g., stocks and flows) from 1 period to the next using a chord diagram provides a richer portrait of contraceptive calendar data that better describes women’s experiences than calculation of discontinuation or failure rates alone. Users showed keen interest in employing the chord diagram but required a short tutorial on the method before being able to take full advantage of the innovative visualization method.

Visualizing contraceptive use, switching, and discontinuation provides a richer portrait of data that better describes women’s experiences than calculating discontinuation or failure rates alone.

Chord diagrams are popular for visualizing migration, another demographic quantity characterized by stocks and flows.^18^ There are similarities in characterizing migration and contraceptive trajectories, including overall rates of and reasons for migration and differences in those rates and reasons by starting point and destination. Given these similarities, the chord diagram should be a similarly versatile and popular tool to help elucidate trends in contraceptive behaviors.

Although the chord diagram is a powerful method to see overall trends in contraceptive behavior in defined populations and time periods, there are documented caveats to using the contraceptive calendar data to track individual contraceptive use including recall and social desirability bias.^1^^,^^9^^,^^19^ Aggregated statistics appear to suffer from less retrospective recall bias,^20^ though this may vary across populations. Since the use of chord diagrams described in this article show aggregated statistics, the data should be no more biased than traditional hazard models. One caveat to consider when viewing a chord diagram of contraceptive use between 2 time points is that the number of users who switch or discontinue may be small, therefore, small/thin arcs should not be overinterpreted. One remedy may be to collapse methods into short- and long-acting and look at churn across higher-order categories rather than by efficacy alone.

The chord diagram described here was the best “out of the box” package in R (*chorddiag* package^13^) to create interactive chord diagrams, making it an easy tool to get up and running for users. The contraceptive calendar tutorials^12^ from the DHS can be used to create event files; at the time of writing this article programs are only available for Stata and SPSS but more programs are being added, which can be loaded into R and manipulated to create interactive chord diagrams. Once users have created event files for the 168 available contraceptive calendars, they could easily switch between surveys in R and create interactive chord diagrams. Users may be able to think of other demographic or public health quantities than can be visualized as flows with a chord diagram.

## CONCLUSION

The chord diagram is a potentially useful way to visualize women’s contraceptive trajectories and can complement a single indicator of the rate of contraceptive discontinuation obtained from hazard models. A chord diagram visualization can be used to augment the hazard of discontinuation calculated using DHS data.

This interactive visualization provides a more dynamic look at contraceptive trajectories that, in the hands of practitioners, researchers, and family planning advocates, can help generate new insights into the contraceptive trajectories that women experience throughout their reproductive lives. The ability to visualize a cohort of women’s contraceptive decision making in detail has important implications for supply chain, health worker development, budget priorities, and contraceptive guidelines. Better knowledge about country-specific trends and questions will allow family planning programmatic investments to reach more women and girls.

## Notes

**Peer Reviewed**

**First Published Online:** December 5, 2019

**Funding:** Bass Connections and Data+ at Duke University
